# Online randomized controlled experiments at scale: lessons and extensions to medicine

**DOI:** 10.1186/s13063-020-4084-y

**Published:** 2020-02-07

**Authors:** Ron Kohavi, Diane Tang, Ya Xu, Lars G. Hemkens, John P. A. Ioannidis

**Affiliations:** 1 Analysis & Experimentation, Microsoft, One Microsoft way, Redmond, WA 98052 USA; 2 Airbnb, 888 Brannan St, San Francisco, CA 94103 USA; 3 Google, 1600 Amphitheatre Parkway, Mountain View, CA 94043 USA; 4 LinkedIn, 950 W Maude Ave, Sunnyvale, CA 94085 USA; 5 Basel Institute for Clinical Epidemiology and Biostatistics, Department of Clinical Research, University Hospital Basel, University of Basel, 4031 Basel, Switzerland; 6 Stanford Prevention Research Center, Department of Medicine, Stanford University School of Medicine, Medical School Office Building, Room X306, 1265 Welch Rd, Stanford, CA 94305 USA; 7 Meta-Research Innovation Center at Stanford (METRICS), Stanford University, Palo Alto, CA 94305 USA; 8 Department of Health Research and Policy, Stanford University School of Medicine, Stanford, CA 94305 USA; 9 Department of Biomedical Data Science, Stanford University School of Medicine, Stanford, CA 94305 USA; 10 Department of Statistics, Stanford University School of Humanities and Sciences, Stanford, CA 94305 USA

**Keywords:** Randomization, A/B tests, Trials, Healthcare decision-making, Online experiments

## Abstract

**Background:**

Many technology companies, including Airbnb, Amazon, Booking.com, eBay, Facebook, Google, LinkedIn, Lyft, Microsoft, Netflix, Twitter, Uber, and Yahoo!/Oath, run online randomized controlled experiments at scale, namely hundreds of concurrent controlled experiments on millions of users each, commonly referred to as A/B tests. Originally derived from the same statistical roots, randomized controlled trials (RCTs) in medicine are now criticized for being expensive and difficult, while in technology, the marginal cost of such experiments is approaching zero and the value for data-driven decision-making is broadly recognized.

**Methods and results:**

This is an overview of key scaling lessons learned in the technology field. They include (1) a focus on metrics, an overall evaluation criterion and thousands of metrics for insights and debugging, automatically computed for every experiment; (2) quick release cycles with automated ramp-up and shut-down that afford agile and safe experimentation, leading to consistent incremental progress over time; and (3) a culture of ‘test everything’ because most ideas fail and tiny changes sometimes show surprising outcomes worth millions of dollars annually.

Technological advances, online interactions, and the availability of large-scale data allowed technology companies to take the science of RCTs and use them as online randomized controlled experiments at large scale with hundreds of such concurrent experiments running on any given day on a wide range of software products, be they web sites, mobile applications, or desktop applications. Rather than hindering innovation, these experiments enabled accelerated innovation with clear improvements to key metrics, including user experience and revenue. As healthcare increases interactions with patients utilizing these modern channels of web sites and digital health applications, many of the lessons apply. The most innovative technological field has recognized that systematic series of randomized trials with numerous failures of the most promising ideas leads to sustainable improvement.

**Conclusion:**

While there are many differences between technology and medicine, it is worth considering whether and how similar designs can be applied via simple RCTs that focus on healthcare decision-making or service delivery. Changes – small and large – should undergo continuous and repeated evaluations in randomized trials and learning from their results will enable accelerated healthcare improvements.

## Background

Every major technology company runs online controlled experiments, often called A/B tests, to gather trustworthy data and make data-driven decisions about how to improve their products. All these controlled experiments are randomized. Companies that make widespread use of this approach include Microsoft [1–3], Google [4, 5], LinkedIn [6–8], Facebook [9], Amazon [10] and Intuit [11]. Much of the methodology used in these online controlled experiments derives from the same family of experimental methods developed in the earlier part of the twentieth century that led to randomized controlled trials (RCT) in medicine [12]. The scale of online controlled experiments has grown dramatically in the last decade, as marginal costs approach zero. In this paper, we share some insights about the evolution and use of A/B tests and derive some key lessons that may be useful for medicine.

It may be possible to translate some of the advantages of online controlled experiments to medicine and invigorate the traditional RCT designs and their applications. In particular, RCTs in medicine are often criticized for being expensive, requiring longer follow-up to obtain reliable answers, and difficult to do. This criticism draws mostly on the paradigm of licensing trials for new medications and biologics, typically done in strictly controlled settings under very specific circumstances. However, a very large number of questions in medicine, health, and healthcare could potentially be answered with simple RCTs at significantly lower cost. Such trials are conducted in a pragmatic fashion and directly address issues of decision-making, such as whether to do or not to do some procedure, test, intervention, information offering, quality improvement, service delivery [13], or management or policy change. They aim to directly compare the effects of choosing option A or option B and outcomes can be collected routinely, for example, obtained from interactions with web sites, mobile applications, and desktop applications, wearable devices or electronic health records, or from reimbursement claims or financial datasets. There are ongoing initiatives aiming to improve the design and affordability of trials or the use of routinely collected data for RCTs [14–16]. Some outcomes may be possible to meaningfully collect very quickly, for example, rehospitalization rates, which is increasingly possible using routinely collected data from electronic health records, administrative data, or registries [13, 16]. In this regard, it would be very useful to learn from the A/B testing experience in technology and allow the medical and healthcare research community to consider whether and how similar designs can be applied in a focused fashion or at massive scale in biomedicine as well.

### The test everything with controlled experiments theme

In the digital world, data is generated and collected at an explosive rate. More than 4 billion of the world’s 7.6 billion population is connected to the internet. The volume and frequency of data production are enormous. For example, Google receives billions of queries every day [17], and along with these queries, terabytes of telemetry data are logged to improve the service. Over the years, technology has also been developed not only to be able to handle the volume and frequency of the data flowing around but also the transfer speed, reliability and security of data. Digital collection of data has become much cheaper and reliable.

At Google, LinkedIn, and Microsoft, where three of the co-authors work, the value of online controlled experiments became clear – tiny changes had surprisingly large impact on key metrics, while big expensive projects often failed. About two-thirds of experiments show that promising ideas that we implemented in products failed to improve the metrics they were designed to change, and this was worse in well-optimized domains such as the search engines [2], where failures were in the range of 80–90%. The humbling results led to a theme of ‘test everything with controlled experiments’ coupled with the idea of testing Minimum Viable Products popularized by Eric Ries in the Lean Startup [18] – the sooner we can get ideas into controlled experiments and thus get objective data, the sooner we can learn and adjust. A motivating example is described in Table 1.

**Table 1:** Example: optimizing after-visit summaries

In the online space, we learned that small changes ranging from making the website faster to changing font colors can meaningfully affect how a user interacts with a product or service, dramatically impacting key metrics, including revenue [3, 19].	
In medicine, with the increasing use of electronic health records, after-visit summaries (AVS) are increasingly used, providing patients with relevant and actionable information similar to traditional patient handouts with a goal of increasing patient compliance and understanding.	
Given that goal:	
• What channel should the AVS use (e.g., paper letter, email, mobile notification) to increase patient engagement?	
• When should the summary be sent? Is there a time of day or day of week (e.g., Friday) when the patient is more likely to engage with the AVS?	
• What text in the message might motivate patients to follow the link? Can we test how to reduce the friction of getting a user to sign-in and view the AVS once they click on a link? How can we reduce the steps required to see the summary?	
• In the AVS summary itself, how is the information presented? Do some layouts improve engagement? Should we present checklists? Reminders? Offer tools (e.g., mobile apps) that can help compliance?	
• There is an increasing focus on the importance of social determinants of health outcomes, so what can we do in terms of sharing the visit summaries with caretakers, be it family members or friends?	
Similar types of questions can be applied in the medical system, and these are exactly the types of questions that online controlled experiments are designed and already used for [20].	

**Fig. 1:**
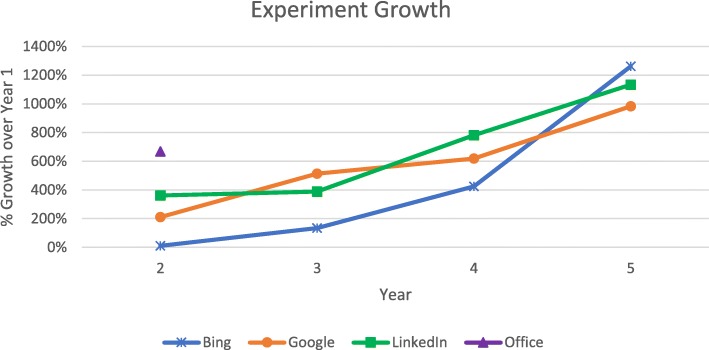
Experimentation growth over the years since experimentation operated at scale of over one new experiment per day

Figure 1 shows how the different organizations scaled experimentation over the years with year 1 being a year where experimentation scaled to over an experiment per day (over 365/year). The graph shows an order of magnitude growth over the next 4 years for Bing, Google, and LinkedIn. In the early years, growth was slowed by the experimentation platform capabilities itself. In the case of Microsoft Office, which just started to use controlled experiments as a safe deployment mechanism for feature rollouts at scale in 2017, the platform was not a limiting factor because of its prior use in Bing, and feature rollouts, run as controlled experiments, grew by over 600% in 2018. Growth slows down when the organization reaches a culture of ‘test everything’ and the limiting factor becomes its ability to convert ideas into code that can be deployed in controlled experiments.

Today, Google, LinkedIn, and Microsoft are at a run rate of over 20,000 controlled experiments/year, although counting methodologies differ (e.g., ramping up the exposure from 1% of users to 5% to 10% can be counted as one or three experiments; an experiment consisting of a control plus two treatments can count as either one or two experiments).

### Phases of technical and cultural change

Software development organizations that start to use controlled experiments typically go through phases of technical and cultural changes as they scale experimentation. Here are key axes on which this evolution at Google, LinkedIn, and Microsoft happened.

#### Scale and statistical power

Firstly, to scale experimentation, the experimentation platform must support the capability of exposing a single user to multiple experiments. Whether the experimentation surface (web site, mobile app, desktop app) has 10,000 monthly active users or 100 million (as Bing, Google, and LinkedIn have), there are never enough users if each user is exposed to just a single experiment. Web sites (like Bing and Google) with multibillion-dollar annual revenues that depend on a single key web page (e.g., the search engine results page, or SERP) imply that we must be able to detect small effects – not detecting a true 0.5% relative degradation to revenue will cost tens of millions of dollars. In the medical literature, looking for such effects would be equivalent to looking for risk ratios of 1.005 or less, which is one order of magnitude lower than the threshold of what are considered ‘tiny effects’ (relative risks < 1.05) [21]. However, this may be very different on a public health level. Here, on a large scale, the impact of tiny effects can be substantial. For example, the effect of fruits and vegetables may be tiny per serving on reducing cancer risk individually (with a HR of 0.999) but substantial at a population level [21].

High statistical power is required, and the way to achieve this is to expose each user to multiple experiments. Because the relationship between the detectable effect and the number of users needed is quadratic [22], the ability to detect an effect twice as small, e.g., 0.25%, requires quadrupling the number of users. For Bing, Google, and LinkedIn, it is common for each experiment to be exposed to over a million users.

If the results are surprising, such as a much larger effect being seen than expected, then the experiment will typically be rerun with tens of millions of users to gain confidence in the results. Both the act of replication and the increased power are important factors in increased trust in the results.

All three companies started with a simple system running experiments on disjoint users, and all switched to concurrent, or overlapping, experiments [2, 4, 7]. A user visiting Bing, Google, or LinkedIn today is exposed to tens of experiments, which may change the user interface, personalization, ranking algorithms, and infrastructure (e.g., improving site speed).

To ensure robustness given the high level of concurrency, mechanisms were developed to prevent interactions (e.g., by declaring constraints or parameters being modified, the system will guarantee disjoint users to those experiments) and nightly tests are sometimes run, which test all pairs of experiments for interactions. A classic example of an interaction has two different experiment treatments wherein each adds a line that pushes the buy button at a retail site down. A user in both treatments experiences a buy button pushed below the ‘fold’ (bottom of screen) and thus add-to-carts drop. In our experience, unexpected interactions in technology are rare and these are addressed by serializing the experiments or, more commonly, by identifying and fixing software issues that show up when users are exposed to multiple experiments.

#### Incremental costs

Secondly, the cost (developer time, data scientist time, hardware resources) of setting up and analyzing experiments is initially high but comes down with scale. As the experimentation platform matures, running and analyzing experiments becomes self-service. For instance, at Google, LinkedIn, and Microsoft, developers, data scientists and product/program managers set up experiments using a browser interface; over 1000 metrics are then computed for each experiment, ranging from various engagement metrics (e.g., pageviews and clicks) to monetization (e.g., revenue and subscription rates) to service metrics (e.g., queries-per-second, latency, and crash rates). It is common that after an experiment is activated, one can get the first read on the experiment impact in minutes for critical metrics. Such near-real-time data pipelines are used to abort egregiously bad experiments or for supporting an experiment to be ramped up from a small percentage of users to a larger one.

Data scientists with statistics and coding background (able to manipulate large amounts of data) are involved in only a small percentage of experiments (e.g., under 5%), where special experiment designs are needed or a deep-dive analysis is required (e.g., two metrics that are normally highly correlated move in opposite directions). As another example of a surprisingly hard problem, some clicks are caused by bots – automated programs that scrape the web site – and should be removed from the analysis as they introduce non-human signals that could skew results or reduce statistical power. At Bing, over 50% of US web traffic is due to bots and the proportion is about 90% in China and Russia; fairly sophisticated mechanisms have been developed to detect bots and remove them.

#### Culture change

Thirdly, when the experimentation platform is no longer limiting the number of experiments (neither technically nor due to costs), the culture changes to the abovementioned ‘test everything with controlled experiments’ mentality. The limiting factor to innovation now becomes the ability to generate ideas and develop the code for them. Software development cycles shrink to enable quick iterations and feedback loops based on the idea of the Minimum Viable Product [18], which means that you build just enough of an idea so that it can test be tested in a controlled experiment and then get feedback and iterate. The key observation is that long development cycles based on the traditional waterfall model often fail to meet their goals due to optimistic assumptions and changing requirements; to paraphrase Helmuth von Moltke, ideas rarely survive contact with customers. Instead, we want to test an idea quickly with real users in a controlled experiment and learn from the results and feedback (mostly implicit, but sometimes explicit through feedback links and survey). Several changes typically happen, as follows:

1. **Release frequency (tempo) improves.** Increasing the frequency of software developments with controlled experiments improves the stability and reliability of software because small changes that are evaluated in isolation allow quick corrections before major maldevelopments have big consequences (e.g., rollbacks) [23, 24]. Release cycles went from 6 months to monthly to weekly to daily, and now at Bing, Google, and LinkedIn, they are made multiple times a day to services and web sites. Experiments on client software, like Microsoft Office, is still limited because, unlike a website, it requires users to update the software on their machines (e.g., PCs or phones). That said, even for client software, release cycles have shrunk from years to weeks, with each release containing hundreds of new features evaluated using controlled experiments.
2. **Agreement on the Overall Evaluation Criterion (OEC) becomes critically important.** An experiment scorecard shows hundreds to thousands of metrics. It is usually easy to find something that improves (or degrades), but the challenge is to come up with a small set of key metrics, ideally a single OEC, to help make tradeoffs. A good OEC captures the organizational long-term objectives but must be based on metrics that are measurable in short-term experiments. Since the OEC is used to determine success (e.g., shipping a change) and consists of one or a few metrics, there is less concern about multiple hypothesis testing. One example of a key component of the OEC is the sessions per user metric [25]; if users are coming more often, it is usually a strong sign that the treatment is useful. The rest of the metrics are used for debugging and understanding why something happened, and these are marked as interesting when the *p* value is low, e.g., < 0.001.
  The reason we can look at so many metrics is that key metrics are broken down by areas. For example, we might be interested in the click-through rate of the page (single metric); to understand the change in this metric, we will show the click-through rate of 20 subareas of the page. In many cases we find that users often have a fixed amount of attention, so there is a conservation of clicks: if one sub-area gets more clicks, it is likely cannibalizing clicks from other sub-areas. In medicine, the issue of competing risks, concurring events, and their capture in combined endpoints integrating the competing components might be the closer analogy to cannibalization of outcomes [26, 27]. Selecting a useful primary outcome(s) is key but not straightforward. Core outcome sets are increasingly developed with input from patients and clinicians to reflect outcomes that cover the long-term objectives of treatment such as the prevention of death, disability, or loss of quality of life [28]. Combined endpoints may integrate several components that may occasionally be competing risks. With a plethora of outcomes, concerns arise about multiplicity [29].
3. **Humbling reality sets in on the value of ideas.** Goals change from ‘ship feature X by date Y’ to ‘improve the OEC by x% over the next year’. Success becomes harder and a humbling reality sets in – most ideas are not as good as we believe [19]. High attrition is similarly common in the development pipeline of medical interventions [30]. Moreover, while many of the more successfully licensed interventions originally have expectations of major benefits, e.g., in survival, most often they settle for improvements in less serious outcomes, e.g., disease progression, without affecting death rates [31].
4. **Evaluation encourages more exploration – breakthrough ideas are discovered.** The safety net afforded by controlled experiments encourages more exploration of ideas that may not be highly prioritized a priori but are easy to code and evaluate. Our experience is that there is no strong correlation between the effort to code an idea and its value. For example, a simple change to ad titles at Bing, which was rated low and took days to code, was worth over $100 M annually [3]. Tweaks to Google’s color scheme, which were shunned by Google’s visual design lead at the time, because he had “*grown tired of debating such minuscule design decisions*” [32] were worth over $200 M annually [33]. In the same way, some medical treatments may have tremendous health effects and are incredibly cheap (e.g., simple diagnostics such as measurement of blood pressure, body temperature or listening to the patient and interventions such as beta-blockers for antihypertensive treatment or antibiotics in sepsis), while high tech interventions that are extremely costly often provide relatively little health gain (e.g., modern oncology treatments [31, 34]).
5. **Incremental progress on long-term goals.** Many long-term improvements are the result of thousands of candidate ideas that are evaluated over multiple iterations. Winners are shipped, losers are modified (given new data and insights from the experiment) or abandoned. It is impressive to see how key metrics have improved over time. This would be the ultimate goal of a learning healthcare system in medicine, where A/B testing might play a crucial role in the continuous evaluation of innovative changes of care [20].

#### Evolution of organizational processes: experimentation maturity on multiple axes

As experimentation matures in an organization [35], the organizational needs evolve, including:

1. **Early indicators and holdout.** While there are metrics that take longer to materialize, such as the retention rate of a paid customer, the desire to iterate quickly usually pushes one to look for early indicators that are then combined with a holdout experiment to see if the long-term metrics differ. Therefore, time to measure is usually a week or a few weeks. For example, a site may give customers a free subscription service trial, and they have 30 days to decide whether they want to subscribe. The customer’s usage and satisfaction of the service during the first few days can be very indicative of whether they will end up paying. In the medical field, such early indicators would be metrics like duration of hospital stay, hospital mortality, complications or 30-day re-admission rates, for example, in clinical trials evaluating different types of surgery.
2. **Near-real-time analysis.** Whereas the initial experimentation system usually produces a scorecard after a day, as reliance on experimentation grows, so does the need for faster scorecards. If there is a bug, a day is too long – too many users are hurt and the development team needs faster feedback. Today, initial scorecards are produced in near-real-time (e.g., every 15 min). While they do not have statistical power to detect the effect we are hoping for, they are sufficient for detecting egregious issues, allowing the platform to abort experiments. Note that, given the large number of scorecards generated, multiple hypothesis issues have to be addressed [2]. The final treatment effect is determined by the final scorecard, usually based on 1–2 weeks of data.
3. **Automated ramp-up.** With near-real-time analysis, it is possible to tradeoff risk versus statistical power. An experiment starts at a small percentage in a single data center, similar to pilot studies in medicine. As discussed above, scorecards are generated in near-real-time and, if certain metrics degrade beyond acceptable limits, the experiment is auto-aborted without the need for human intervention. If after several hours no key metric degrades, the experiment auto-ramps to a higher percentage of users and at multiple data centers.
4. **Heterogeneous treatment effects are provided in scorecards.** Rather than focus just on the average treatment effect, the scorecard also highlights interesting segments, where the treatment effect is different than the average. For example, a browser version (say Internet Explorer 8) may behave differently, leading to a discovery that JavaScript code failed in that setting; in other cases, low performance in a country or market may be due to poorly localized text. The key is that hypotheses develop and experiments start to target segments of users. In contrast to typically underpowered subgroup analyses in medical clinical trials, these experiments are highly powered with enough users that the segments are big enough for reliable statistical analyses.
5. **Trustworthiness.** With so many experiments running, there is an obvious concern for lack of trustworthiness and false positive results. We exercise multiple tests to identify scenarios that would indicate a problem [36] such as, for instance, skewed assignments. For example, suppose the experiment design calls for equal assignment to control treatment and that the actual number of control users is 821,588 and of treatment users is 815,482, and thus the ratio is 50.2% instead of 50%. The system would flag this as a sample-ratio-mismatch and declare the experiment result invalid, as the *p* value for such a split is 1.8x10^–6^. For dealing with multiple hypothesis testing problems, we replicate experiments. In areas like search relevance, teams are measured on the sum of treatment effects of a single key metric and, because many experiments are run, once a positive result is found, it is rerun, and the replication run determines the actual credit the team gets. The replication effect is unbiased, while the first run may have found an exaggerated effect [37].
6. **Institutional memory.** With tens of thousands of experiments run every year, it is important to highlight surprising results (both failures and successes). Some are published in conferences [19] or websites [38], but internal presentations and documents are important for cross-pollination.

A summary of the lessons for medicine learned in the technology field is given in Table 2.

**Table 2:** Lessons learned

• The philosophy of ‘test everything with controlled experiments’, i.e., the consistent and systematic implementation and integration of evaluation into the entire development and application of treatments and innovations is equivalent to the philosophy of ‘randomize the first patient’ principle in medicine, that was introduced more than 40 years ago. However, this has met much more resistance in medicine	
• Technological advances and the availability of large-scale data makes it tempting to abandon randomized trials, while randomization is precisely what has turned out to be so useful for the most successful technology companies	
• Rather than hindering innovation, randomized trials fostered improvements to products and revenue	
• The most innovative technological field has recognized that systematic series of randomized trials with numerous failures of the most promising ideas leads to sustainable improvement	
• Various parallels exist in the application of randomization, including the importance of selecting the best evaluation criterions (outcome measures)	
• Even tiny changes should ideally undergo continuous and repeated evaluations in randomized trials and learning from their results may be indispensable also for healthcare improvement	

### Similarities and dissimilarities with medical RCTs

Given their large sample sizes and scale, large scale A/B tests in technology allow addressing some additional design implementation issues that would have been difficult to address in traditional medical RCTs, which have rarely very large sample sizes to date. Some interesting topics are covered in Table 3. Several of the features of A/B experiments discussed above can be adopted in RCTs in medicine and do not necessarily require a very large scale; the principles described here are already used in healthcare, although rarely. For example, Horwitz et al. describe a “*rapid-cycle randomized testing*” system that has been established in NYU Langone Health in the US and allowed to complete 10 randomized A/B tests, involving several hundred to several thousands of patients, within 1 year, with annual costs of $350,000 [20]. By testing various interventions that are introduced in routine care every day in many places in the world, and typically without randomized evaluation, they were able to determine what really works and systematically improved healthcare in their hospital: “*We now know with confidence that changing the text of a provider-targeted prompt to give tobacco cessation counseling in an office produces a significant increase in rates of medication prescriptions and that changing just a few sentences in telephone outreach scripts can both shorten telephone calls and increase rates of appointments for annual examinations. We have also learned that our postdischarge telephone calls have made no difference in rates of readmission or patient-experience ratings, that our appointment-reminder letters were completely ineffective, and that our community health worker program was inadvertently targeting patients who were unlikely to benefit*” [20].

**Table 3:** Methodological issues that can be overcome in online experiments to date, difficult in traditional medical RCTs, but potentially relevant in future large-scale medical RCTs

There are usually many quality checks that are feasible in the online space with large-sample A/B tests. Here are a few examples:	
• **Checks on randomization**: If the experiment design is for a ratio of one-to-one (equally sized control and treatment) then deviations in the actual ratio of users in an experiment likely indicate a problem. With large numbers, a ratio smaller than 0.99 or larger than 1.01 for a design that called for 1.0 likely indicates a serious issue. This simple test has identified numerous issues in experiments, many of which looked either great or terrible initially and invoked Twyman’s law (“Any figure that looks interesting or different is usually wrong”) for us [39].	
• **Bias assessment with A/A tests**: A/A test is the same as an A/B test, but the treatment and control users receive identical experience (the same UI, or the same ranking algorithms etc.), thus differences measured by the experimental procedures reflect chance or bias. Because the null hypothesis is true by design in A/A tests, statistically significant differences for each metric should happen at about 5% when using a *p* value cutoff of 0.05. We can run a large number of A/A tests easily, and a higher or lower A/A failure rate for metrics would happen when the normality or independent and i.i.d. assumptions (i.e. independent and identically distributed data) are violated. A/A tests are also used to ensure reasonable balance between treatment and control users. They can be very effective at identifying biases, especially those introduced at the platform level. For example, we can use A/A tests to identify carry-over effect (or residual effect), where previous experiments would impact subsequent experiments run on the same users [25].	
• **Re-randomization or post-experiment adjustment.** Randomization, while it is a great technique to remove confounding factors, is not the most efficient at times. For example, we may have more engaged users in treatment than in control just by chance. While stratification is a common technique used to improve balance across strata, it can be expensive to implement efficiently during the sampling phase. One effective approach is to check the balance of key metrics using historical data and then re-randomize using a different hash ID if the difference between the treatment and the control is too large. For instance, Microsoft has created a ‘seed finder’ that can try hundreds of seeds for the hash function to see which one leads to a difference that is not statistically significant [25]. Another approach is to apply adjustment during the analysis phase, using post-stratification or CUPED [40]. Netflix [41] has a nice comparison paper on some of these approaches.	

The most desirable features of A/B experiments are their large-scale and low cost, which are commensurate with the tradition of large simple trials [42] and the emerging interest in pragmatic trials [43, 44]. Lower costs would allow to test more and other interventions and provide better evidence on thus far understudied healthcare questions [13, 16]. Online administration is also commensurate with the emerging efforts to perform point-of-care randomization [45]. The principles of ongoing, routine data collection for outcomes has parallelisms to the concept of using routinely collected data, e.g., from electronic health records, to fuel RCT datasets with proper outcomes [46].

There is less emphasis in medical RCTs on performing multiple RCTs at the same time and engaging the same participants in multiple concurrent RCTs. However, besides the traditional factorial designs [47], there is some literature, especially on lifestyle, about performing multiple concurrent parallel randomizations [48].

A major difference between A/B testing in technology and medical RCTs is their time horizon. Many RCTs in biomedicine would require longer follow-up, often much longer than that afforded by technology A/B trials. However, if a data collection system is in place (e.g., electronic health records), such data collection may be automated and real-time assembly of data would be feasible. Moreover, in acute medical treatment settings, there are many patient-relevant and economically important outcomes that can be collected in the short time frame, such as duration of hospital stay, admission to intensive care or re-admission rates.

Ethical implications are different between the technology field and medicine. There is a push towards having more trials that are simple and which compare usual care modifications that are already implemented somewhere or would be implemented anyway without ethical approval [49]. The evaluation of minor usual care modifications may be seen more as quality improvement than research [50] and using randomization alone may not necessarily define an evaluation as research [20].

Finally, the A/B concept may be particularly attractive for healthcare services, management, and improvement interventions, where most of the current research pertains to non-randomized before–after studies and interrupted time series. Essentially, each digital interaction, use of diagnostic software or algorithm, or electronic decision aid could and maybe should be evaluated and optimized in a randomized experiment.

## Summary and discussion

Randomization is recognized as a powerful tool that technology companies successfully use at extremely large scale to improve their products and increase revenue. Not only the origins of the methods are similar in the technology world and the medical field, there are also many parallels in possible applications. However, the consistent and systematic implementation and integration into the entire development and application cycles have no such parallel in the biomedical world. The development and ongoing evaluation of new interventions as well as the many interfaces between users and providers of healthcare are far from optimal. There is substantial potential to improve health if these can be optimized.

Recently, criticism of randomized trials in medicine seems to be growing. Technological advances and the availability of large-scale data makes it tempting to abandon randomization, while randomization is precisely what has turned out to be so useful for the most successful technology companies. The technology world has demonstrated, on several occasions, that promising ideas in the vast majority of cases do not prove useful once they have been tested in online controlled experiments. While this has repeatedly been shown also for various cases in the medical world and various estimates of the extent of the problem exist, technology companies can objectively measure the failure rate and directly assess the true value of randomization. When most of the promising, plausible changes of practice turned out to be wrong, and even tiny changes of usual practice had substantial impact on key outcomes, a philosophy of ‘test everything with controlled experiments’ was established. Rather than hindering innovation; it fostered improvements to products and revenue.

Perhaps this is the most important lesson to be learned by the medical world. The most innovative technological field has recognized that systematic series of randomized experiments with numerous failures leads to sustainable improvement of the products. Even tiny changes should ideally undergo continuous and repeated evaluations in randomized experiments and learning from their results may be indispensable also for healthcare improvement.

## Data Availability

Not applicable.
